# A New Electro-Optical Switch Modulator Based on the Surface Plasmon Polaritons of Graphene in Mid-Infrared Band

**DOI:** 10.3390/s19010089

**Published:** 2018-12-28

**Authors:** Ming Cai, Shulong Wang, Bo Gao, Yindi Wang, Tao Han, Hongxia Liu

**Affiliations:** Key Laboratory for Wide Band Gap Semiconductor Materials and Devices of Education, School of Microelectronics, Xidian University, Xi’an 710071, China; cm9999787@163.com (M.C.); zzflys@163.com (B.G.); wangyindi4213@126.com (Y.W.); 15639119745@163.com (T.H.)

**Keywords:** surface plasmon polaritons, graphene, electro–optical switch modulator

## Abstract

In this paper, a new electro-optical switch modulator based on the surface plasmon polaritons of graphene is proposed. An air–graphene-substrate–dielectric structure is adopted in the modulator. In this structure, the graphene is considered as a film of metal whose thickness tends to be infinitesimal. By changing the external voltage, the boundary conditions can be changed to decide whether the surface plasmon polariton waves can be excited in mid-infrared band. Because of this effect, the structure can be used as an electro–optical switch modulator, whose modulation depth is about 100% in theory. Finally, the 3 dB bandwidth (~34 GHz) and the energy loss (36.47 fJ/bit) of the electro–optical switch modulator are given, whose low energy loss is very suitable for engineering applications.

## 1. Introduction

Surface plasmon polaritons (SPPs) [1,2,3,4,5] refer to the surface transmission mode of the electromagnetic field, which is produced by the collective movement of electrons in metals when the incident light is irradiated to the metal surface. When they have an equal frequency resonance, the transversal magnetic field and transversal electric field (TM and TE) polarized state can be excited to transmit along metal and media boundary surface [6,7]. Based on the transmission characteristic, the surface plasmon waveguide can bind the light field within the range of subwavelength magnitude. Therefore, it is possible to make micro-nano optoelectronic devices by using the properties of the surface polarization wave, which is conducive to the realization in miniaturization of optoelectronic integrated devices. In recent decades, the research on surface plasmon polaritons relies on precious metal materials represented by gold and silver. Using these precious metal materials, researchers have proposed a large number of waveguide [8,9,10,11,12] structures that can bind the light field at the nanometer level. These excellent waveguide structures can be applied to integrating photoelectric modulator and so on.

Graphene [13,14,15,16], a new type of two-dimensional material, has attracted much attention from many researchers for its various good properties. In graphene’s crystal structure, carbon atoms are arranged periodically in a two-dimensional plane in a hexagonal honeycomb, and three valence electrons can form SP^2^ bonds. For this crystal structure, graphene has excellent electrical and optical properties. Specifically, in a wide spectral range, the optical absorption rate of graphene with intrinsic monolayer thickness is 2.3%, which is 50 times the same thickness as gallium arsenide. Based on the strong absorption effect of graphene on light, the light absorption can be adjusted by changing the voltage [17] and create an electro–optical modulator with graphene. In addition, graphene can be used to change the boundary conditions of the medium interface, which transforms the excitation of the surface plasmon polaritons waves in the whole structure. Basing on this theory, a new electro–optical switch modulator is realized.

An electro–optical switch [18,19,20,21] modulator [22,23,24,25] refers to the transmission and cutoff of mode light in the devices through the change of external voltage. This mode of light propagation can be regarded as a switch in essence.

The mid-infrared band is a vital band, which has important application in different fields. For example, it can be used in sensing, environmental monitoring, and other places. Optoelectronic devices have many advantages, which include the greater plasma dispersion effects and the ease of manufacture in current technology.

Many researchers have used the tunability of graphene to achieve electro–optical modulators, but the large volume and low modulation depth become the common problem for these modulators. To solve these problems, an air–graphene-substrate–dielectric structure is proposed. It makes use of the tunability of graphene and realizes the transmission or cutoff of the medium exciter wave in the whole structure. Theoretically, this modulator can achieve the modulation depth of 100%, which cannot be obtained by the traditional electro–optical modulator. Finally, the 3 dB bandwidth of the electro–optical switch modulator and energy loss of the switch state are also given. The modulator proposed here is very suitable for engineering application because of the low energy loss.

## 2. Structure Design

**Figure 1:**
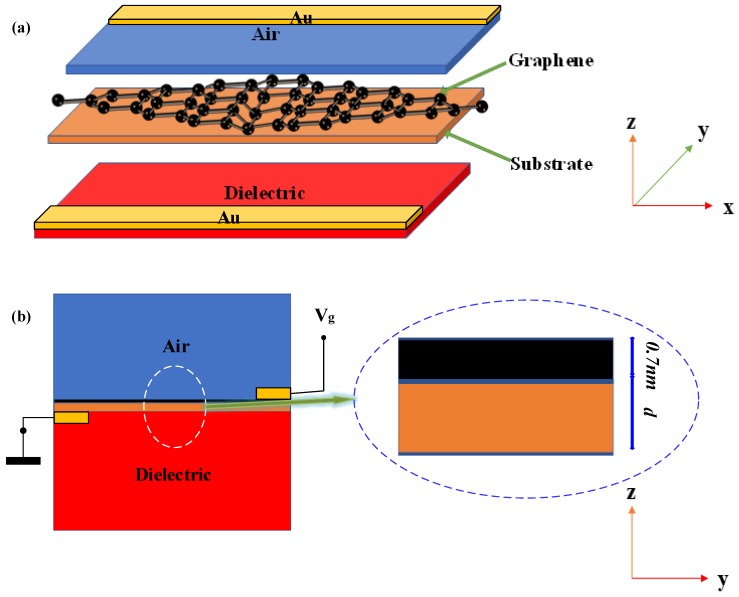
The stereograph and sectional view of this modulator: (**a**) 3D layout waveguide structure, (**b**) cross-section structure.

The new electro–optical switch modulator is composed of an air–graphene-substrate–dielectric structure. Concretely speaking, it uses graphene-substrate as the thin film metals between the interface of air and dielectric (the substrate and dielectric use the same materials). And for the electrode Au, it is placed in the position shown in Figure 1.

This design can change the electrical conductivity of graphene by changing the voltage, to regulate the transmission and cutoff of surface plasmon polaritons waves. Finally, the aim of electro–optical modulation is achieved.

## 3. Methodology and Models

The principle involved in this paper is mainly divided into two parts. One part is the change of the relationship between voltage and the graphene photoconductivity. Another part is the excitation of the graphene material to the dielectric interface surface plasmon polaritons waves.

The relationship between voltage and graphene photoconductivity can be obtained by using the relationship between the chemical potential (Fermi level) and the cooper equation obtained from the plate model.

For the graphene, it’s carrier concentration n_0_ can be given by Equation (1).

(1) $$n_{0}=\frac{Q}{S\cdot e}$$

where *Q* is the charge of the capacitor, *e* indicates the amount of electron charge and the size is 1.6 × 10^−19^ C. *S* represents the area of the plate capacitor. And the *Q* can be given by Equation (2).

(2) $$Q=C(V+V_{0})$$

where *V* refers to the voltage applied externally. *V*_0_ is related to the Fermi level of graphene when it is not applied to voltage, which is usually to take 0 V.

**Figure 2:**
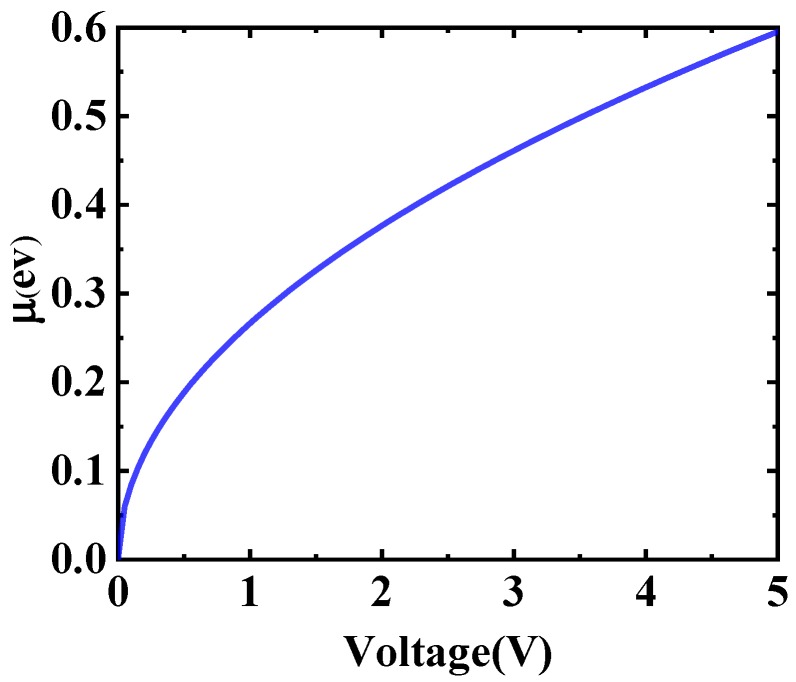
The image of chemical potential as a function of voltage (The μ represents the chemical potential).

Figure 2 shows the relationship between voltage and chemical potential (Fermi level). According to the Equations (1) and (2), the relation between them can be given by Equation (3).

(3) $$\mu =\hbar v_{f}\sqrt{\pi \cdot n_{0}}=\hbar v_{f}\sqrt{\pi \frac{\varepsilon_{0}\varepsilon_{r}}{d\cdot e}(V+V_{0})}$$

Among them, *ħ* represents the reduced Planck’s constant whose size is about 1.055 × 10^−34^ J·s. The *V_f_* indicates the Fermi velocity of graphene, which is 1.1 × 10^6^ m/s. *ε*_0_ refers to the dielectric constant in free space, and *ε_r_* is the relative dielectric constant of the substrate material. *d* represents the thickness of graphene and substrate.

**Figure 3:**
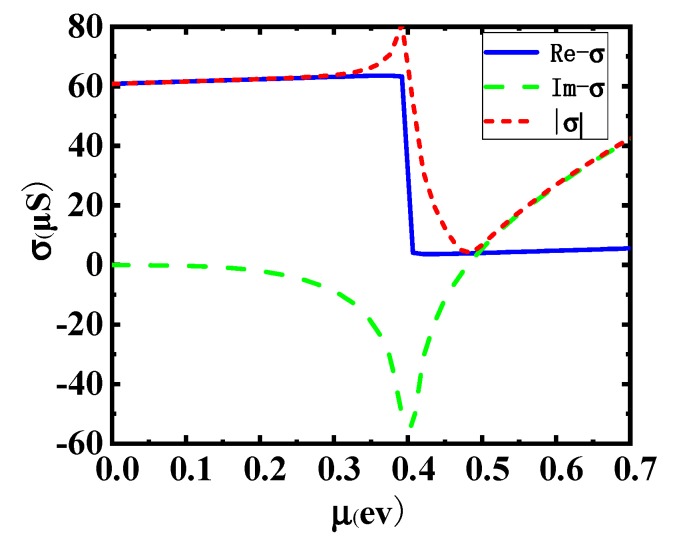
The image of conductivity as a function of chemical potential.

Figure 3 shows the relation between graphene chemical potential and electrical conductivity, and the relation between them can be obtained by Equation (4).

$$\sigma_{\mathrm{int}ra}=\sigma_{0}\frac{4\mu }{\pi }\frac{1}{\hbar \tau_{1}-i\hbar w}$$

$$\sigma_{\mathrm{int}\mathrm{er}}^{\prime }=\sigma_{0}(1+\frac{1}{\pi }\arctan\frac{\hbar w-2\mu }{\hbar \tau_{2}}-\frac{1}{\pi }\arctan\frac{\hbar w+2\mu }{\hbar \tau_{2}})$$

$$\sigma_{\mathrm{int}er}^{\prime \prime }=-\sigma_{0}\frac{1}{2\pi }\ln\frac{{\left(2\mu +\hbar w\right)}^{2}+\hbar^{2}\tau_{2}^{2}}{{\left(2\mu -\hbar w\right)}^{2}+\hbar^{2}\tau_{2}^{2}}$$

(4) $$\sigma =\sigma_{\mathrm{int}ra}+\sigma_{\mathrm{int}er}^{\prime }+\sigma_{\mathrm{int}er}^{\prime \prime }$$

Among them, *w* refers to the angle frequency of incident light. *τ*_1_ and *τ*_2_ indicate the intra-band and inter-band lag time of graphene. Here it is taken 10 fs and 1.2 ps respectively. In addition, the *σ*_0_ is equal to πe^2^/2h.

By this way, the relationship voltage and the conductivity of graphene can be obtained.

The boundary conditions of Maxwell equations are given in Equation (5).

$$D_{n2}-D_{n1}=\rho$$

$$E_{t2}-E_{t1}=0$$

$$B_{n2}-B_{n1}=0$$

(5) $$H_{t2}-H_{t1}=J_{s}$$

In Equation (5), *ρ* represents the volume charge of the medium interface, and *J_s_* indicates the surface current size of the medium interface.

The graphene can be seen as a layer current. So, combining the Maxwell equations, the surface plasmon dispersion relation in TM mode can be seen in Equation (6).

(6) $$\frac{\varepsilon_{1}}{k_{1}}+\frac{\varepsilon_{2}}{k_{2}}+\frac{i\sigma }{w\varepsilon_{0}}=0$$

Among them, *k*_1_ and *k*_2_ represent the longitudinal wave vectors of light up and below the interface. *σ* refers to the conductivity of graphene. According to the knowledge of surface plasmon wave, the real part of *k*_1_ and *k*_2_ must be greater than 0 (otherwise the surface plasmon waves do not exist), to ensure that the surface plasmon waves decrease from the interface to the medium internal. For the conductivity of graphene, the positive or negative values of the imaginary part in it will affect the positive and negative values of *k*_1_ and *k*_2_. Through this change, the excitation and cutoff of the surface plasmon waves in the TM mode can be controlled very easily.

In the same way, the dispersion relationship of the graphene in TE mode can be obtained by Equation (7).

(7) $$k_{1}+k_{2}=iw\sigma$$

From the Equation (7), the state of surface plasmon polariton waves can be controlled by changing the conductivity of graphene.

To sum up these two parts, the changing of external voltage can directly influence the transmission or cutoff about surface plasmon polariton waves in this new structure. In this way, a new type of electro–optical switch modulator is proposed.

## 4. Results and Discussion

**Figure 4:**
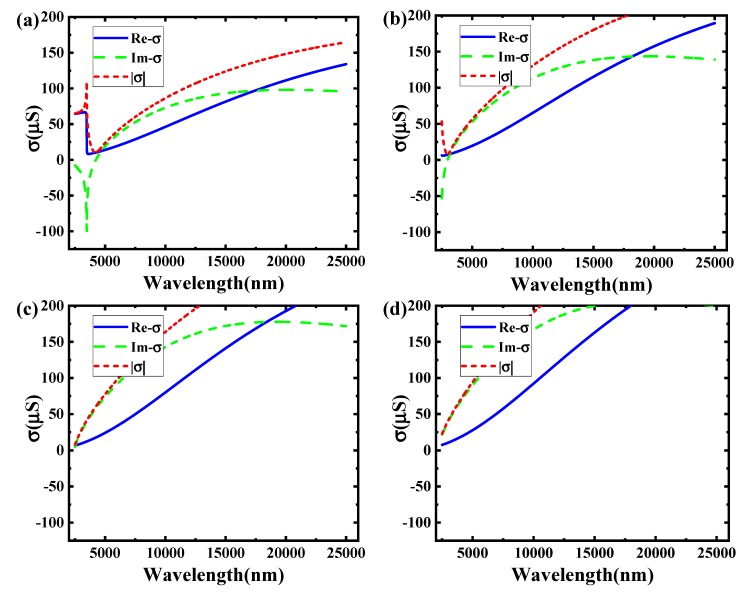
The image of conductivity as a function of incident wavelength in different voltage. (**a**) 0.5 V; (**b**) 1 V; (**c**) 1.5 V; (**d**) 2 V.

Figure 4 shows the relationship between the incident wavelengths and the graphene conductivity in different voltages at the boron nitride substrate thickness of 10 nm. It corresponds to the voltage in 0.5 V, 1 V, 1.5 V, and 2 V.

According to Figure 4, it is obvious that changing conductivity of graphene can be obtained by transforming the voltages. Comparing these pictures, the imaginary parts of graphene conductivity become larger as the voltage increases. In the voltages of 0.5 V and 1 V, the imaginary of graphene conductivity has both positive value and negative value. In the voltages of 1.5 V and 2 V, the imaginary of conductive all become positive values. It is illustrated that some incident wave bands cannot be transmitted in TM mode. On the contrary, it can be all transmitted in the 1.5 V and 2 V.

This phenomenon is the opposite in TE mode. In the voltages of 0.5 V and 1 V, some incident wave bands can be spread in TE mode. But for the voltages in 1.5 V or 2 V, TE mode cannot be spread in the mid-infrared band (2500 nm~25,000 nm).

**Figure 5:**
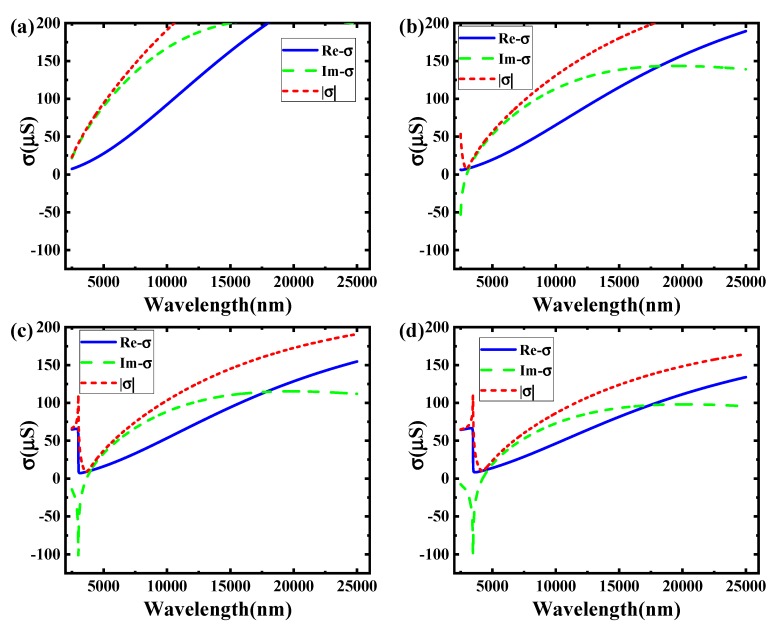
The image of conductivity as a function of incident wavelength in different substrate thicknesses. (**a**) 5 nm; (**b**)10 nm; (**c**) 15 nm; (**d**) 20 nm.

Figure 5 shows the relationship between the incident wavelengths and the graphene conductivity in 1 V at the boron nitride substrate. The corresponding thickness is respective 5 nm, 10 nm, 15 nm, and 20 nm.

It can be observed from Figure 5 that the substrate thickness has a significant influence on the conductivity of graphene. The imaginary of conductivity is completely greater than 0 μS in 5 nm, which is different in 10 nm, 15 nm, and 20 nm. It illustrates that the incident light waves cannot propagate the TE mode in 5 nm. For the other substrate thickness, the incident lights can be transmitted in some incident bands.

For the TM mode, all incident waves in Mid-infrared band can spread in 5 nm, which cannot be achieved in 10 nm, 15 nm, and 20 nm.

**Figure 6:**
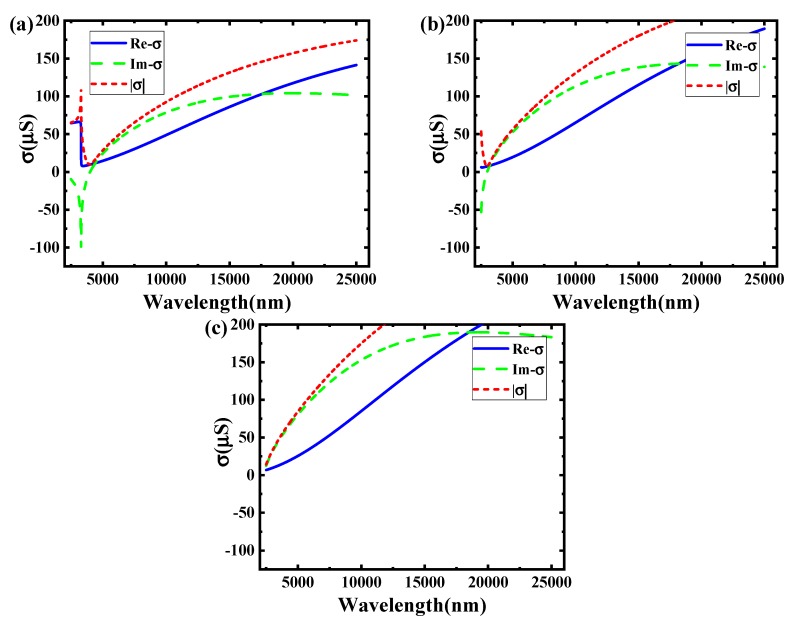
The image of conductivity as a function of incident wavelength in different substrate materials. (**a**)SiO_2_; (**b**) BN; (**c**) Si.

Figure 6 shows the relationship between the incident wavelengths and the graphene conductivity in 1 V at the different substrates in 10 nm thickness. It corresponds to the different substrates in SiO_2_, BN, and Si.

According to Figure 6, the changing of substrate material has a strong influence on the conductivity of graphene. For the material Si, all imaginary for graphene conductivity is greater than 0 μS, which could not be seen in the other material. It shows that the material Si cannot spread the incident waves in TE mode. But some incident waves could be propagated in BN and Si.

On the contrary, any Mid-infrared band waves can be spread in TM mode with Si. But the other materials only spread the incent waves in some mid-infrared bands.

The two situations (1 V and 1.5 V) in Figure 4 can be used for an electro–optical modulator to achieve the modulation function. For the 2500 nm incident wavelength, the imaginary of graphene conductivity is about −50 μS in 1 V and 1 μS in 1.5 V.

According to Equation (7), when the external voltage is 1 V, the incident waves can spread in TE mode. It cannot spread in 1.5 V for the same mode. Therefore, it can be used as an electro–optical switch modulator.

According to Equation (8), the bandwidth of the electro–optical modulator can be given as:

(8) $$f_{3dB}=\frac{1}{2\pi RC}$$

Among them, *R* represents the resistance of the whole structure. *C* refers to the capacitance of the whole structure. After the calculation, the 3 dB bandwidth is about 34 GHz in this structure.

According to Equation (9), the energy loss of the electro–optical modulator can be given as:

(9) $$E=\frac{1}{4}CV_{P}^{2}$$

where *V_p_* represents the peak value of the external voltage. Using the 1 V and 1.5 V as the two switch voltages of this modulator, the energy loss is about 36.47 fJ/bit during one cycle.

## 5. Conclusions

In this paper, a new electro–optical switch modulator based on surface plasmon polaritons of graphene was proposed. It used the tunable of graphene and the properties about surface plasmon polaritons to achieve the control of external voltage to the transmission of incident waves. This control way can be applied to design for an electro–optical switch modulator, which has 100% modulation depth, in theory. It cannot be obtained by traditional electro–optical modulator. In addition, it has very low energy loss (~36.47 fJ/bit), which is very adapt to products in engineering.
